# Atherogenic index of plasma and the risk of advanced subclinical coronary artery disease beyond traditional risk factors: An observational cohort study

**DOI:** 10.1002/clc.23450

**Published:** 2020-08-20

**Authors:** Ki‐Bum Won, Mi‐Hee Jang, Eun Ji Park, Hyung‐Bok Park, Ran Heo, Donghee Han, Hyuk‐Jae Chang

**Affiliations:** ^1^ Division of Cardiology, Ulsan University Hospital University of Ulsan College of Medicine Ulsan South Korea; ^2^ Division of Cardiology, Severance Cardiovascular Hospital, Yonsei University College of Medicine Yonsei University Health System Seoul South Korea; ^3^ Division of Cardiology, Asan Medical Center University of Ulsan College of Medicine Seoul South Korea; ^4^ Medical information Center Ulsan University Hospital Ulsan South Korea; ^5^ Division of Cardiology Catholic Kwandong University International St. Mary's Hospital Incheon South Korea; ^6^ Division of Cardiology, Hanyang University Seoul Hospital Hanyang University College of Medicine Seoul South Korea; ^7^ Division of Cardiology, New York‐Presbyterian Hospital and Weill Cornell Medical College New York New York USA

**Keywords:** atherogenic index of plasma, atherosclerosis, coronary computed tomography angiography, risk assessment, serum marker

## Abstract

**Background:**

Atherogenic lipoprotein profile of plasma is an important risk factor for atherosclerosis. The atherogenic index of plasma (AIP) has been suggested as a novel marker for atherosclerosis.

**Hypothesis:**

AIP is a useful marker of advanced subclinical coronary artery disease (CAD) in subjects without overt renal dysfunction.

**Methods:**

A total of 6928 subjects with estimated glomerular filtration rate > 60 mL/minutes/1.73 m^2^ evaluated by coronary computed tomography angiography (CCTA) for health check‐up were included. The relation of AIP to advanced CAD (heavy coronary calcification, defined as coronary artery calcium score [CACS] >100 or obstructive coronary plaque [OCP], defined as plaque with >50% stenosis) was evaluated.

**Results:**

All participants were stratified into four groups based on AIP quartiles. The prevalence of CACS >100 (group I [lowest] 4.7% vs group II 7.0% vs group III 8.8% vs group IV 10.0%) and OCP (group I 3.7% vs group II 6.4% vs group III 8.8% vs group IV 10.9%) (all *P* < .001) increased with elevating AIP quartiles. Higher AIP (per 0.1 unit increase) was associated with an increased risk of CACS >100 (odds ratio [OR] 1.057, 95% confidence interval (CI) 1.010 to 1.106, *P* = .017; relative risk (RR) 1.048, 95% CI 1.009‐1.089, and *P* = .015) and OCP (OR 1.079, 95% CI 1.033‐1.127, *P* = .001; RR 1.069, 95% CI 1.031‐1.108, *P* < .001) after adjusting for age > 60 years, male sex, hypertension, diabetes mellitus, dyslipidaemia, obesity, and proteinuria.

**Conclusion:**

AIP is independently associated with advanced subclinical CAD beyond traditional risk factors.

## 1 INTRODUCTION

Atherogenic lipoprotein profile of plasma is an important risk factor for atherosclerosis. The atherogenic index of plasma (AIP) has been suggested as a marker of plasma atherogenicity based on its strong and positive association with cholesterol esterification rates, lipoprotein particle size, and remnant lipoproteinaemia.^1^, ^2^, ^3^ In addition, previous data have shown that AIP is more closely related to cardiovascular (CV) risk than individual lipoprotein cholesterol fractions or other atherogenic indices.^4^, ^5^, ^6^ However, data on the association of AIP with the advanced subclinical coronary artery disease (CAD) beyond traditional risk factors are limited in clinical practice.

Coronary artery calcium score (CACS) has been regarded as a useful marker of coronary atherosclerosis because it represents the degree of atheromatous plaque burden.^7^, ^8^ Recently, coronary computed tomography angiography (CCTA) has been used as an effective and noninvasive tool to assess the presence of plaques, severity of stenosis, and subtypes of plaque in coronary arteries with a robust ability to predict major adverse CV events.^9^, ^10^, ^11^ Thus, this study evaluated the association of AIP with advanced subclinical CAD in subjects without overt renal dysfunction using CCTA.

## 2 METHODS

### 2.1 Study population and design

This observational, retrospective, single‐center registry consisted of 8648 consecutive participants evaluated by CCTA for health check‐up with 64‐slice multidetector computed tomography between January 2004 and April 2009 at Severance CV hospital.^12^ Of them, 1720 subjects were excluded because of (a) age < 30 years (n = 69), (b) overt renal dysfunction defined as glomerular filtration rate (GFR) estimated by modification of diet in renal disease (MDRD) method <60 mL/minutes/1.73 m^2^ (n = 958), and (c) insufficient medical records for traditional risk factor and AIP calculation or poor image quality (n = 693). As a result, 6928 participants were finally included in the present study. The study protocol was approved by the ethics committee of severance CV Hospital, and informed consent for the procedure was obtained from each participant. Patients or the public were not involved in the design, conduct, reporting, or dissemination of our research.

Medical history of hypertension, diabetes mellitus, and dyslipidaemia was systematically acquired. Height, body weight, and blood pressure (BP) were measured during visits. Height and weight measurements were obtained while the subjects wore light clothing without shoes. Body mass index (BMI) was calculated as weight in kilograms divided by the square of height in meters. After the participants rested for ≥5 minutes, the BP was measured at the right arm using an automatic manometer with an appropriate cuff size. Serum levels of total cholesterol, triglycerides, high‐density lipoprotein cholesterol (HDL‐C), low‐density lipoprotein cholesterol (LDL‐C), glucose, and creatinine were measured after a minimum of 12 hours fasting period. The AIP was calculated as the base 10 logarithm of the ratio of the triglycerides to HDL‐C concentrations.^1^ The kidney function was ascertained by estimated GFR calculated using the MDRD formula.^13^ Hypertension was defined as systolic BP ≥140 mmHg and/or diastolic BP ≥90 mmHg or treatment with antihypertensive agents. Diabetes mellitus was defined as treatment with hypoglycaemic agents or insulin, or fasting glucose ≥126 mg/dL. Dyslipidaemia was defined as serum levels of total cholesterol ≥240 mg/dL, LDL‐C ≥ 130 mg/dL, HDL‐C ≤ 40 mg/dL, triglycerides ≥150 mg/dL, and/or treatment with lipid lowering agents. Obesity was defined as BMI ≥25 kg/m^2^ based on the cutoffs for Asian population. Proteinuria was defined based on the sex‐specific urinary albumin/creatinine ratio.^14^, ^15^ Age ≥ 60 years, male sex, hypertension, diabetes mellitus, dyslipidaemia, obesity, and proteinuria were considered as traditional risk factors in the present study. All procedures were in accordance with the ethical standards of the institutional research committee (Giessen, AZ.: 127/16) and with the 1964 Helsinki declaration and its later amendments or comparable ethical standards.

### 2.2 CCTA protocol

**FIGURE 1:**
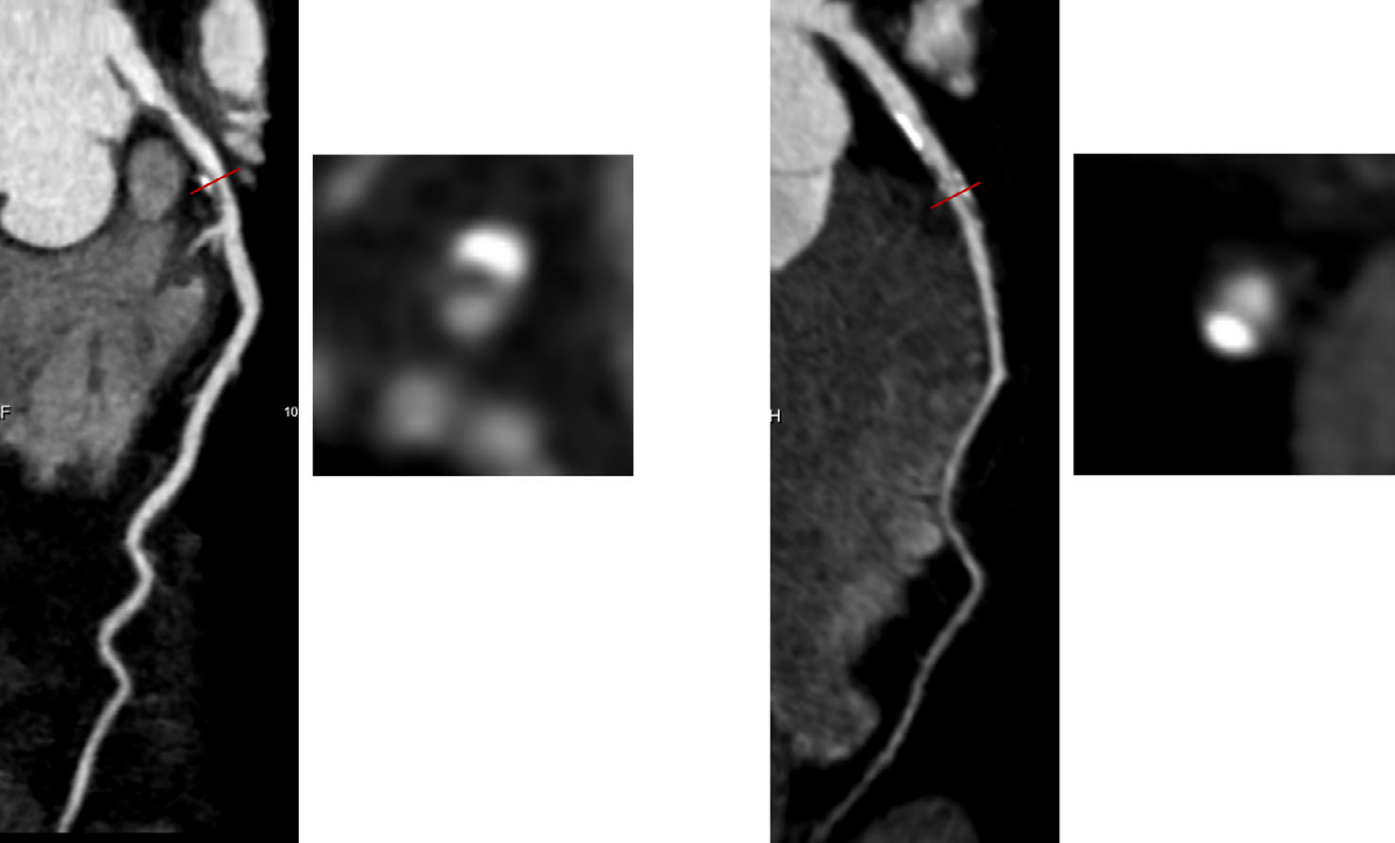
Representative CCTA images. CCTA, coronary computed tomography angiography

During CCTA examination, subjects with an initial heart rate > 65 beats/minutes received a single oral dose of 50 mg metoprolol tartrate (Betaloc, Yuhan, Seoul, Korea) 1 to 2 hours before the examination if beta blocking agents were not contraindicated. A 64‐slice scanner (Sensation 64; Siemens Medical Systems, Forchheim, Germany) was used for examination. First, a nonenhanced prospective electrocardiogram (ECG)‐gated scan was obtained for the measurement of CACS with the following parameters: rotation time of 330 ms, slice width of 3.0 mm, slice collimation of 0.6 mm, tube current of 50 mA, tube voltage of 120 kV, and table feed/scan of 18 mm. Subsequently, CCTA was performed using retrospective ECG gating with the following scan parameters: rotation time of 330 ms, slice collimation of 64 × 0.6 mm, tube voltage of 100 to 120 kV, tube current of 600 to 800 mA depending on patient size, table feed/scan of 3.8 mm, and pitch factor of 0.2. ECG‐based tube current modulation was applied to 65% of the R‐R interval. A real‐time bolus‐tracking technique was used to trigger the initiation of the scan. The total estimated average radiation dose for the multislice protocol was 10.9 ± 1.9 mSv. Contrast enhancement was achieved with 60 mL iopamidol (370 mg iodine/mL, Iopamiro; Bracco, Milan, Italy) injected at 5 mL/s, followed by an injection of 30 mL diluted contrast, and then 30 mL saline at 5 mL/s using an injector (Envision CT; Medrad, Indianola, Pennsylvania). The images were evaluated by two experienced cardiac radiologists. CACS, which was measured using the scoring system described by Agatston et al,^16^ was categorized into four groups based on the following scores: 0, 1 to 10, 11 to 100, and >100. Coronary plaques were defined as structures ≥1 mm^2^ within or adjacent to the vessel lumen, which were clearly distinguishable from the lumen and the surrounding pericardial tissue. Plaques without calcium were classified as noncalcified, those with calcified tissue involving ≥50% of the plaque area (density > 130 HU) were classified as calcified, and those with <50% calcium were classified as mixed plaques.^17^ Two outcome variables were selected for identifying the advanced CAD: (a) CACS >100 and (b) obstructive coronary plaque (OCP) defined as coronary plaque with ≥50% luminal narrowing. Representative CCTA images are presented in Figure 1.

### 2.3 Statistical analysis

Continuous variables were expressed as mean ± SD. Categorical variables were presented as absolute values and proportions. To compare the characteristics among the AIP quartiles, one‐way analysis of variance was used for continuous variables, and the χ^2^‐test or Fisher's exact test was used for categorical variables, as appropriate. Univariate logistic regression analysis was performed to identify the association between clinical variables and coronary atherosclerotic parameters. Multiple logistic regression analysis was used to identify the predictive value of AIP for advanced CAD. The forced entry method was used to enter independent variables into the multivariate regression analysis model. Receiver operating characteristic (ROC) curve analysis with Youden index was conducted to determine an optimal cutoff of AIP for predicting advanced CAD. We then compared the predictive significance of the optimal AIP cutoff for advanced CAD after considering traditional risk factors. All statistical analyses were performed with the Statistical Package for the Social Sciences version 19 (IBM Corp., Armonk, New York) and SAS version 9.1.3 (SAS Institute Inc., Cary, North Carolina). A *P*‐value <.05 was considered significant for all analyses.

## 3 RESULTS

### 3.1 Baseline characteristics

**FIGURE 2:**
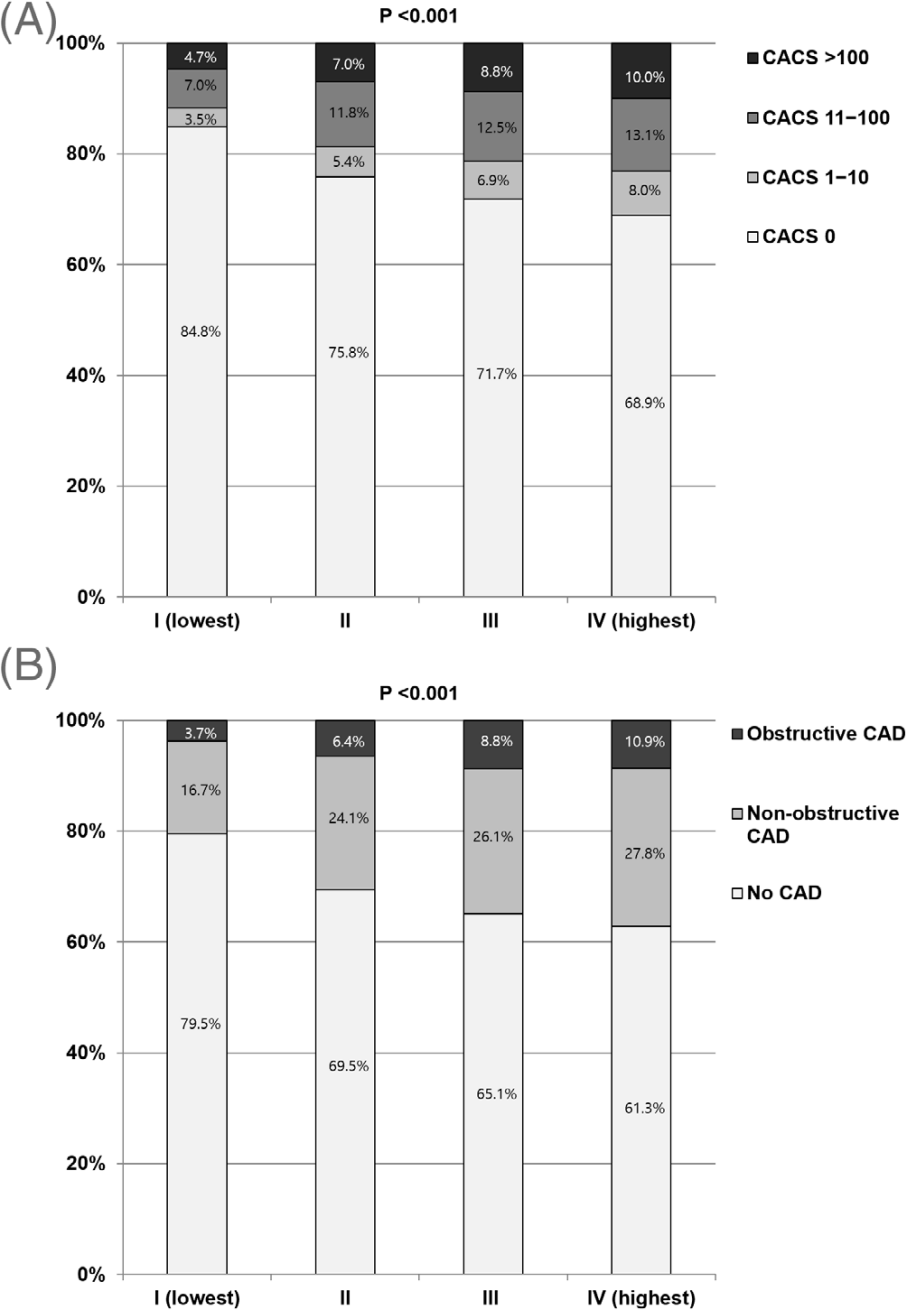
Comparison of CACS and OCP according to AIP quartiles. AIP, atherogenic index of plasma; CACS, coronary artery calcium score; CAD, coronary artery disease; OCP, obstructive coronary plaque

The mean age of participants was 52.0 ± 9.7 years and 3977 (57.4%) of them were men. The prevalence of hypertension, diabetes mellitus, dyslipidaemia, obesity, and proteinuria was 28.8%, 11.2%, 53.8%, 38.0%, and 6.6%, respectively. The CACS >100 and OCP was observed in 7.6% and 7.4% of subjects, respectively. All participants were categorized into four‐groups based on the AIP quartiles. The ranges of the AIP in stratified groups of I (lowest), II, III, and IV (highest) were 0.60 to 0.14, 0.15 to 0.35, 0.36 to 0.55, and 0.56 to 1.79, respectively. Systolic and diastolic BP values, serum levels of total cholesterol, triglycerides, glucose, and creatinine, albumin/creatinine ratio, and the prevalence of male sex, hypertension, diabetes mellitus, dyslipidaemia, obesity, and proteinuria significantly increased with increasing AIP quartiles. In contrast, the mean level of HDL‐C significantly decreased with increasing AIP quartiles (Table 1). The proportion of categorical CACS and OCP according to AIP quartiles is presented in Figure 2. The prevalence of coronary plaque sub‐types according to AIP quartiles is presented in Supplementary Figure 1.

**TABLE 1:** Baseline characteristics of included subjects

	Quartiles of AIP				*P*
	I (lowest) (n = 1764) −0.60 − 0.14	II (n = 1792) 0.15‐0.35	III (n = 1690) 0.36‐0.55	IV (highest) (n = 1682) 0.56‐1.79	
Age, years	50.3 ± 9.4	52.9 ± 9.7	53.1 ± 9.8	51.9 ± 9.5	<.001
Male, n (%)	628 (35.6)	979 (54.6)	1123 (66.4)	1247 (74.1)	<.001
Systolic BP, mmHg	124.8 ± 14.6	125.8 ± 14.6	127.0 ± 14.2	127.4 ± 15.2	.012
Diastolic BP, mmHg	78.2 ± 9.3	78.2 ± 9.4	79.3 ± 9.7	79.8 ± 9.7	.004
BMI, kg/m^2^	22.7 ± 2.8	24.0 ± 2.8	24.8 ± 2.7	25.4 ± 2.7	<.001
Hypertension, n (%)	340 (19.3)	507 (28.3)	570 (33.7)	579 (34.4)	<.001
Diabetes mellitus, n (%)	109 (6.2)	184 (10.3)	208 (12.3)	275 (16.3)	<.001
Dyslipidemia, n (%)	326 (18.5)	637 (35.5)	1084 (64.1)	1681 (99.9)	<.001
Obesity, n (%)	321 (18.2)	623 (34.8)	756 (44.7)	931 (55.4)	<.001
Total cholesterol, mg/dL	191.8 ± 33.0	195.7 ± 36.4	198.8 ± 35.9	200.3 ± 36.8	<.001
Triglyceride, mg/dL	64.8 ± 15.6	99.4 ± 19.6	140.1 ± 27.1	241.3 ± 111.6	<.001
HDL‐C, mg/dL	66.2 ± 12.5	55.1 ± 9.5	49.3 ± 8.3	42.7 ± 7.6	<.001
LDL‐C, mg/dL	104.7 ± 28.0	115.8 ± 30.0	120.9 ± 30.9	113.7 ± 30.1	<.001
Glucose, mg/dL	97.4 ± 17.2	100.1 ± 22.2	102.3 ± 22.7	107.5 ± 32.2	<.001
Creatinine, mg/dL	0.94 ± 0.16	0.98 ± 0.17	1.00 ± 0.17	1.02 ± 0.17	<.001
GFR, mL/min/1.73 m^2^	76.4 ± 11.3	77.1 ± 11.8	77.5 ± 11.1	78.2 ± 11.3	<.001
Albumin creatinine ratio	10.0 ± 22.4	10.0 ± 17.2	14.2 ± 62.6	25.3 ± 128.4	<.001
Proteinuria, n (%)	90 (5.5)	109 (6.6)	106 (6.7)	152 (9.8)	<.001

*Note*: Values are presented as the mean ± SD or number (%).

Abbreviations: AIP, atherogenic index of plasma; BMI, body mass index; BP, blood pressure; HDL‐C, high‐density lipoprotein cholesterol; LDL‐C, low‐density lipoprotein cholesterol.

### 3.2 Clinical variables and the risk of advanced CAD

The association of clinical variables with CACS >100 and OCP is presented in Table 2. Age, male sex, hypertension, diabetes mellitus, dyslipidaemia, obesity, and proteinuria were significantly associated with an increased risk of both CACS >100 and OCP. With regard to the association between AIP and the risk of advanced CAD, the risk of CACS >100 and OCP was significantly higher in group II, group III, and group IV compared with that in group I, respectively.

**TABLE 2:** Association of clinical variables with CACS >100 and obstructive plaque

	CACS >100		OCP	
	OR (95% CI)	*P*	OR (95% CI)	*P*
Age, years	1.136 (1.123‐1.148)	<.001	1.099 (1.088‐1.110)	<.001
Male	2.203 (1.803‐2.692)	<.001	2.029 (1.662‐2.478)	<.001
Hypertension	2.733 (2.283‐3.271)	<.001	2.445 (2.038‐2.932)	<.001
Diabetes mellitus	3.948 (3.217‐4.845)	<.001	3.303 (2.672‐4.083)	<.001
Dyslipidemia	1.483 (1.234‐1.782)	<.001	1.906 (1.573‐2.309)	<.001
Obesity	1.331 (1.112‐1.592)	.002	1.387 (1.157‐1.663)	<.001
Proteinuria	1.756 (1.303‐2.366)	<.001	1.560 (1.145‐2.127)	.005
AIP quartiles				
I	1	–	1	–
II	1.553 (1.167‐2.068)	.003	1.764 (1.294‐2.406)	<.001
III	1.990 (1.507‐2.628)	<.001	2.469 (1.833‐3.327)	<.001
IV	2.281 (1.736‐2.997)	<.001	3.141 (2.350‐4.197)	<.001

Abbreviations: AIP, atherogenic index of plasma; CACS, coronary artery calcium score; CAD, coronary artery disease; CI, confidence interval; OCP, obstructive coronary plaque; OR, odds ratio.

### 3.3 Predictive value of AIP for advanced CAD

In ROC curve analysis, the optimal AIP cutoff for predicting CACS >100 and OCP were 0.28 (sensitivity 70.5%, specificity 42.6%, area under curve (AUC) 0.581, 95% CI 0.556‐0.606, *P* < .001) and 0.33 (sensitivity 47.9%, specificity 68.5%, AUC 0.617, 95% confidence interval (CI) 0.593‐0.641, *P* < .001), respectively (Supplementary Figure 2). After considering traditional risk factors, the predictive significance of AIP levels greater than individual optimal cutoff for CACS >100 (AIP >0.28 vs AIP > 0.28 with traditional risk factors AUC 0.566 vs 0.813, *P* < .001) and OCP (AIP >0.33 vs AIP >0.33 with traditional risk factors AUC 0.589 vs 0.762, *P* < .001) incrementally improved (Supplementary Figure 3).

### 3.4 Impact of AIP on the risk of advanced CAD beyond traditional risk factors

To identify the association of AIP with the risk of CACS >100 and OCP, consecutive adjustment of individual traditional risk factors was performed. An increase of AIP level (per 0.1 unit) was associated with an increased risk of CACS >100 (odds ratio [OR] 1.057, 95% (CI) 1.010‐1.106, *P* = .017; relative risk (RR) 1.048, 95% CI 1.009‐1.089, *P* = .015) and OCP (OR 1.079, 95% CI 1.033‐1.127, *P* = .001; RR 1.069, 95% CI 1.031‐1.108, *P* < .001) after adjusting for traditional risk factors (Table 3). Regarding the association of individual components of AIP with the risk of CACS >100 and OCP after adjusting for traditional risk factors, an increase of triglycerides level was associated with an increased risk of CACS >100; however, there was no significant association between triglycerides level and the risk of OCP. In contrast, an increase of HDL‐C level was associated with a decreased risk of OCP, but not of CACS <100 (Supplementary Table 1).

**TABLE 3:** Impact of AIP (per 0.1 unit increase) on CACS >100 and OCP

	CACS >100				OCP			
	OR (95% CI)	*P*	RR (95% CI)	*P*	OR (95% CI)	*P*	RR (95% CI)	*P*
Model 1	1.101 (1.068‐1.135)	<.001	1.092 (1.064‐1.122)	<.001	1.147 (1.112‐1.182)	<.001	1.133 (1.104‐1.162)	<.001
Model 2	1.080 (1.044‐1.118)	<.001	1.068 (1.037‐1.100)	<.001	1.131 (1.093‐1.169)	<.001	1.114 (1.083‐1.146)	<.001
Model 3	1.057 (1.010–1.106)	.017	1.048 (1.009‐1.089)	.015	1.079 (1.033–1.127)	.001	1.069 (1.031–1.108)	<.001

*Note*: Model 1: unadjusted, Model 2: adjusted for age > 60 years and male sex, and Model 3: adjusted for age > 60 years, male sex, hypertension, diabetes mellitus, dyslipidaemia, obesity, and proteinuria.

Abbreviations: AIP, atherogenic index of plasma; CACS, coronary artery calcium score; CAD, coronary artery disease; CI, confidence interval; OCP, obstructive coronary plaque; OR, odds ratio; RR, relative risk.

## 4 DISCUSSION

In the present study, a significant association was observed between AIP and the risk of advanced subclinical CAD in subjects beyond traditional risk factors. Recently, CCTA has been widely used for the comprehensive evaluation of coronary atherosclerosis, including lesion location, severity, and plaque characteristics in clinical practice.^18^ The CONFIRM (Coronary CT angiography evaluation for clinical outcomes: An international multicenter registry) studies previously revealed that the severity of CAD as revealed by CCTA has a strong predictive value for adverse clinical outcomes.^19^, ^20^ We considered a CACS >100 an indicator of advanced coronary artery calcification based on the facts that (1) Nasir et al.^21^ reported that the frequency of a CACS >100 in the Asian population is significantly lower than that in Western populations and (2) the overall proportion of CACS >100 was 7.6% in our participants. Additionally, we defined OCP as a plaque with ≥ 50% stenosis because 1) all participants received a CCTA examination for health check‐up and (2) the prevalence of OCP was 7.4% in this study.

High levels of the triglycerides‐to‐HDL‐C ratio have been associated with obesity and metabolic syndrome.^22^, ^23^ This might be related to the fact that hypertriglyceridemia stimulates the activity of cholesteryl ester transfer protein, which exchanges triglycerides from triglyceride‐rich lipoproteins for cholesteryl esters from high‐ and LDLs^24^; triglycerides enrichment of high‐ and LDLs particles renders them better substrates for lipolysis by hepatic lipase, resulting in HDL catabolism and elimination and the formation of more numerous, denser LDLs particles. Previously, the very large database of lipids‐4 (VLDL‐4) study showed that a higher ratio of triglycerides to HDL‐C was associated with an increasingly atherogenic lipid phenotype, characterized by higher remnant lipoprotein particle cholesterol along with higher nonHDL‐C and LDL‐C density.^3^ Millán et al^25^ suggested the simultaneous use of triglycerides‐to‐HDL‐C ratio is more useful than isolated lipid values as it more closely reflects the complex interactions of lipoprotein metabolism and can better predict plasma atherogenicity. Based on this strong evidence, the AIP has been suggested as an effective marker for plasma atherogenicity.

Recently, several studies demonstrated that AIP is closely related to the coronary atherosclerosis in specific clinical conditions.^26^, ^27^ However, large sample size data on the value of AIP for advanced subclinical CAD over traditional risk factors in general population are limited. In this observational cohort study with cross‐sectional design, we could identify the close association of AIP with CACS >100 and OCP in 6928 Korean adults evaluated by CCTA for health check‐up. In addition, the predictive significance of AIP for advanced subclinical CAD improved after considering traditional risk factors together. Further prospective longitudinal studies are necessary to confirm the role of AIP for assessing the degree of coronary atherosclerosis in clinical practice.

Several limitations to the study should be acknowledged. First, this was a retrospective, cross‐sectional, and observational study. Thus, selection bias might be present. Second, potential roles of other environmental risk factors and the diet or exercise in subclinical atherosclerosis were not evaluated. Finally, this study included only the Korean adults, which may limit the generalization of obtained results to other populations. Despite these limitations, this study is unique in that an independent association was identified between AIP and advanced subclinical coronary atherosclerosis beyond traditional risk factors in an Asian population. Furthermore, the present study investigated the association of AIP with coronary plaque subtypes.

In conclusion, elevated AIP levels are significantly associated with a higher risk of advanced subclinical CAD as revealed by CCTA in Korean adults with near‐normal renal function even after adjusting for traditional risk factors. With consideration of traditional risk factors together, AIP may be an effective predictive marker for advanced coronary atherosclerosis in general population without overt renal dysfunction.

## CONFLICT OF INTERESTS

The authors declare no potential conflict of interest.

## AUTHOR CONTRIBUTIONS

All authors have made substantial contributions. Ki‐Bum Won and Eun Ji Park performed the statistical analysis. Hyuk‐Jae Chang contributed to the data acquisition. Ki‐Bum Won, Mi‐Hee Jang, Eun Ji Park, Hyung‐Bok Park, Ran Heo, Donghee Han, JHL, and Hyuk‐Jae Chang contributed to the data interpretation. Ki‐Bum Won and Mi‐Hee Jang drafted the manuscript. Hyuk‐Jae Chang critically revised the manuscript. All authors read and approved the final manuscript. All authors also gave final approval and agree to be accountable for all aspects of work ensuring integrity and accuracy.

## Supporting information


**Supplementary figure 1** Prevalence of coronary plaque subtypes according to AIP quartiles
AIP, atherogenic index of plasma

**Supplementary figure 2** Optimal cutoff of AIP for CACS >100 and OCP
AIP, atherogenic index of plasma; AUC, area under curve; CACS, coronary artery calcium score; CI, confidence interval; OCP, obstructive coronary plaque

**Supplementary figure 3** Comparison of ROC models related to the cutoffs of AIP for predicting advanced subclinical CAD
AIP, atherogenic index of plasma; AUC, area under curve; CACS, coronary artery calcium score; CAD, coronary artery disease; OCP, obstructive coronary plaque; ROC, receiver operating characteristic

**Supplementary table 1** Association of individual components of AIP with CACS >100 and OCP beyond traditional risk factors
