# Supplementary figures and images for: Atherogenic index of plasma and the risk of advanced subclinical coronary artery disease beyond traditional risk factors: An observational cohort study
Source: Clin Cardiol. 2020 Aug 20;43(12):1398–404. doi: 10.1002/clc.23450 (PMC7724231; doi:10.1002/clc.23450)

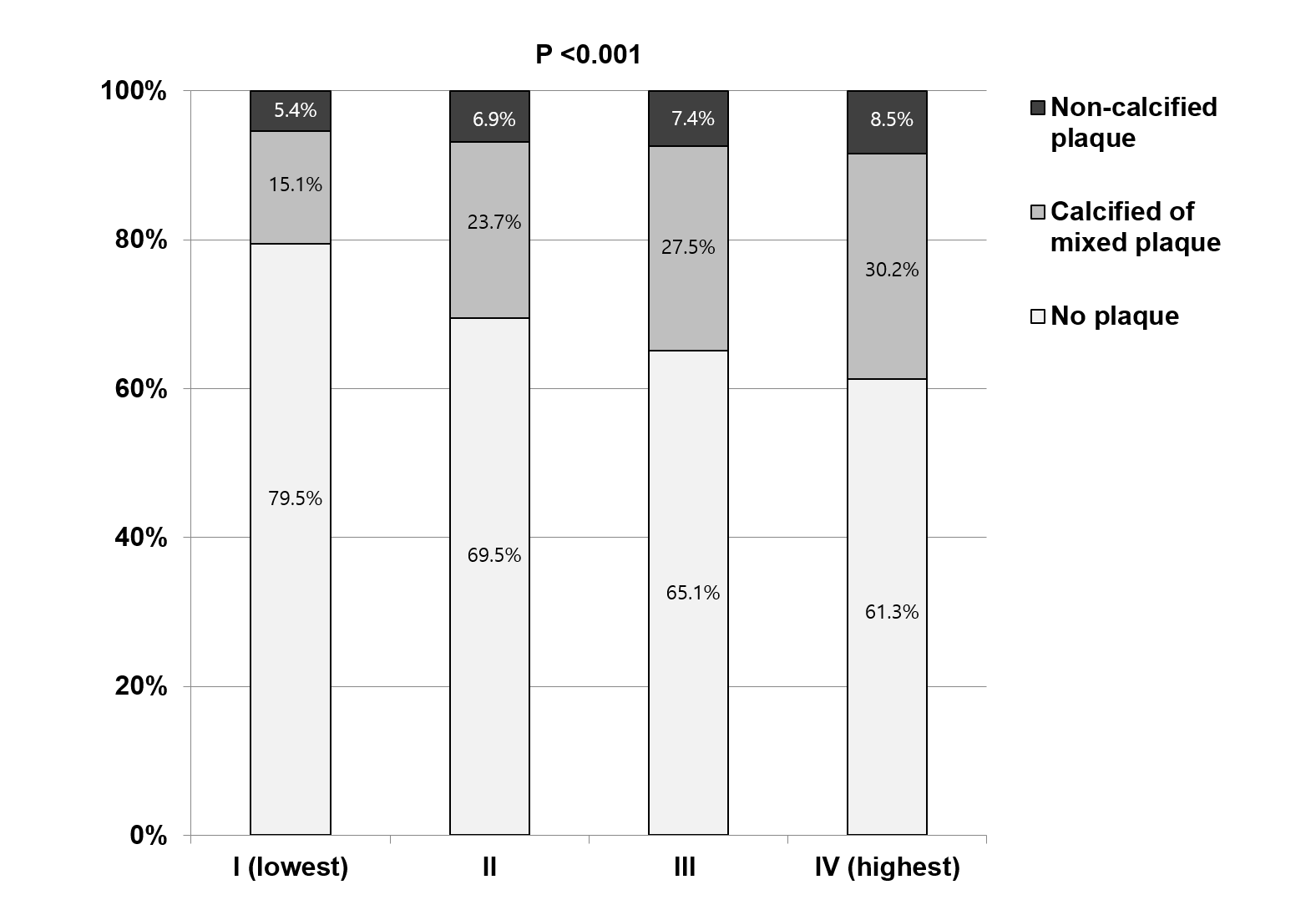

Supplement: Supplementary file 1 — Supplementary figure 1 Prevalence of coronary plaque subtypes according to AIP quartiles AIP, atherogenic index of plasma [file CLC-43-1398-s001.tif]

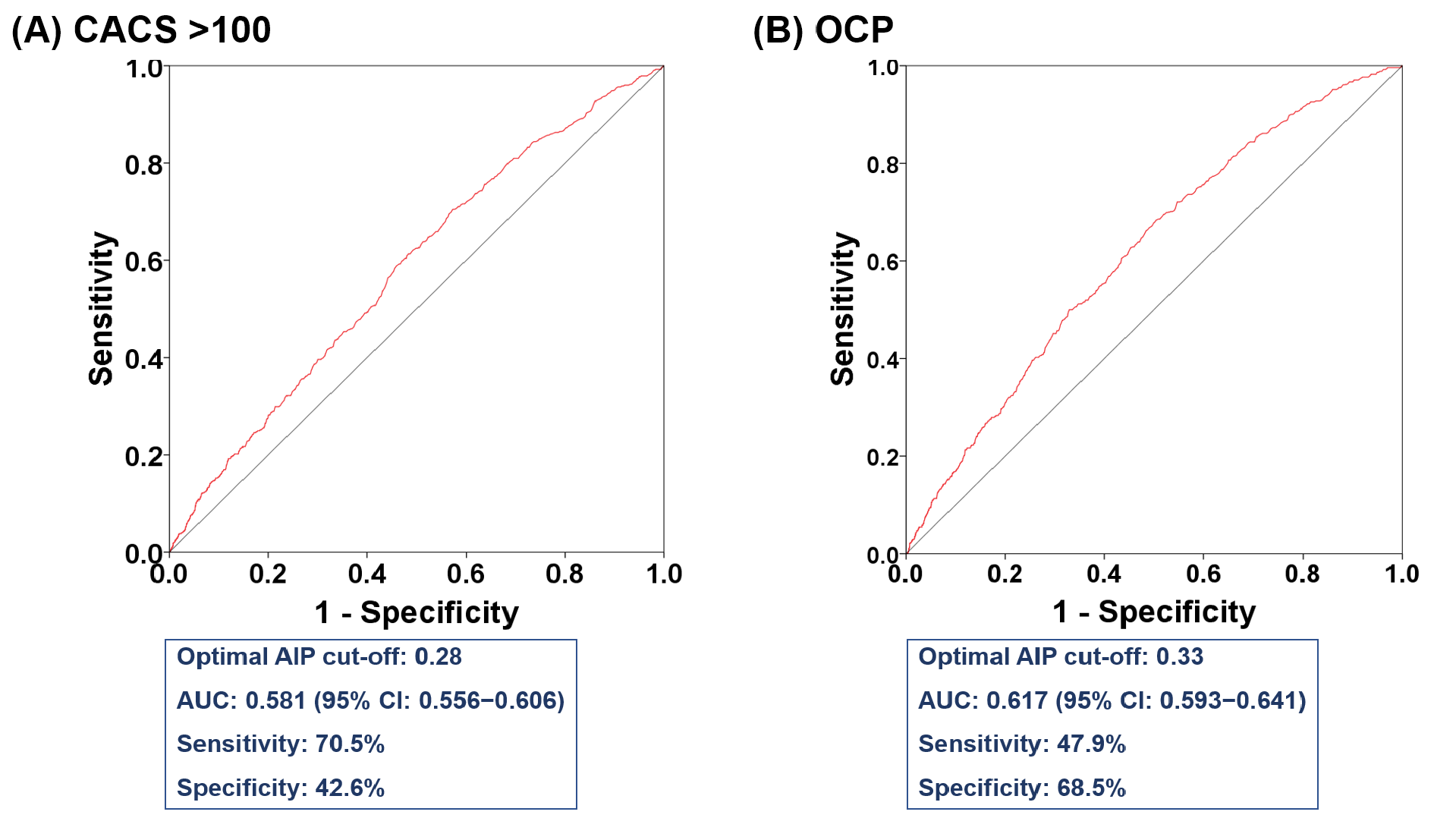

Supplement: Supplementary file 2 — Supplementary figure 2 Optimal cutoff of AIP for CACS >100 and OCP AIP, atherogenic index of plasma; AUC, area under curve; CACS, coronary artery calcium score; CI, confidence interval; OCP, obstructive coronary plaque [file CLC-43-1398-s002.tif]

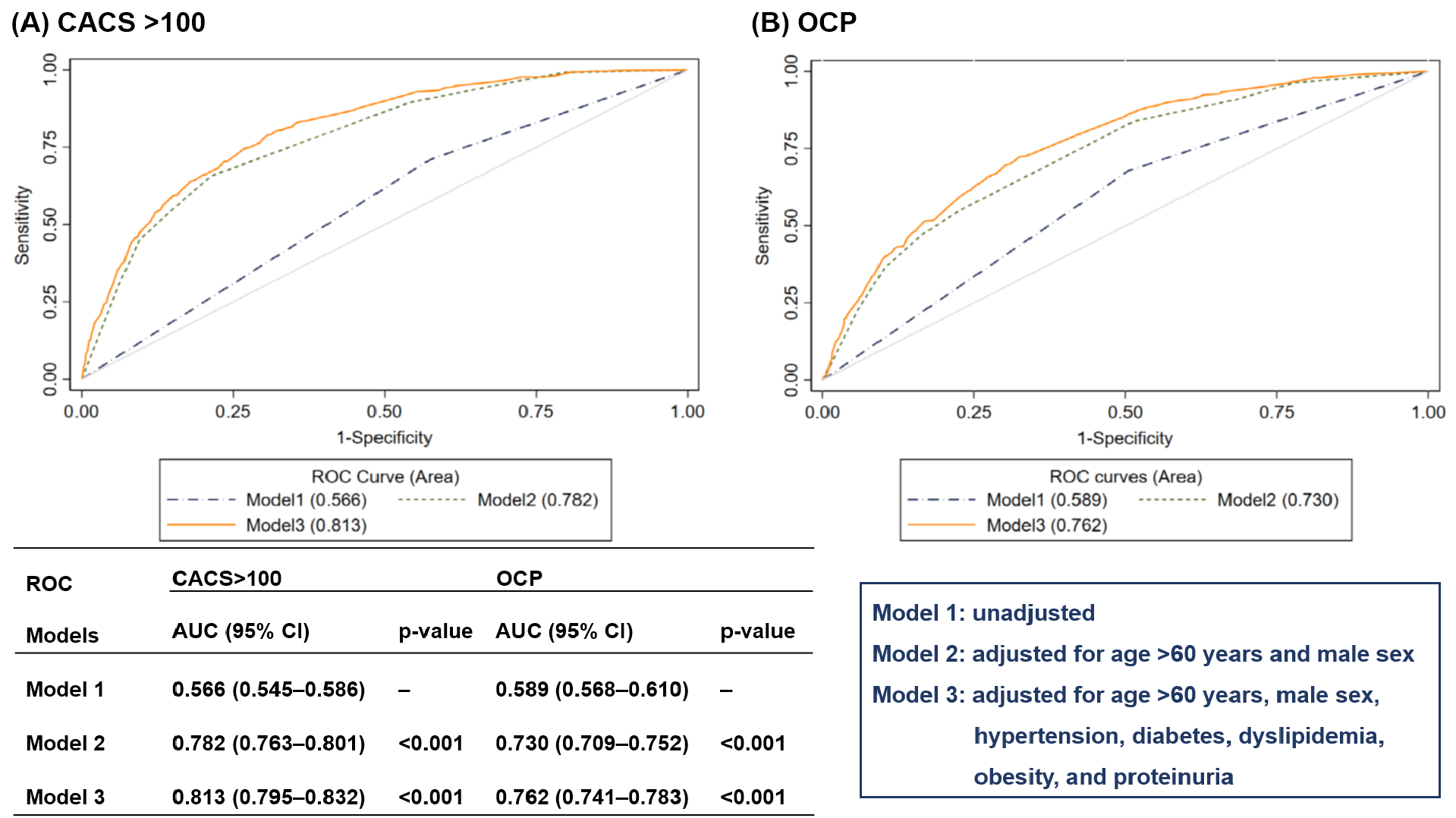

Supplement: Supplementary file 3 — Supplementary figure 3 Comparison of ROC models related to the cutoffs of AIP for predicting advanced subclinical CAD AIP, atherogenic index of plasma; AUC, area under curve; CACS, coronary artery calcium score; CAD, coronary artery disease; OCP, obstructive coronary plaque; ROC, receiver operating characteristic [file CLC-43-1398-s003.tif]
