# Supplementary material for: Atherogenic index of plasma and the risk of advanced subclinical coronary artery disease beyond traditional risk factors: An observational cohort study
Source: Clin Cardiol. 2020 Aug 20;43(12):1398–404. doi: 10.1002/clc.23450 (PMC7724231; doi:10.1002/clc.23450)
Supplement: Supplementary file 4 — Supplementary table 1 Association of individual components of AIP with CACS >100 and OCP beyond traditional risk factors [file CLC-43-1398-s004.docx]

**Supplementary table 1**. Association of individual components of AIP with CACS >100 and OCP beyond traditional risk factors

|  | CACS >100 | | OCP | |
| --- | --- | --- | --- | --- |
|  | OR (95% CI) | P | OR (95% CI) | P |
| Triglyceride, per 1mg/dL increase |  |  |  |  |
| Model 1 | 1.002 (1.001–1.003) | <0.001 | 1.002 (1.001–1.003) | <0.001 |
| Model 2 | 1.001 (1.000–1.002) | 0.043 | 1.001 (1.000–1.002) | 0.077 |
| HDL-C, per 1mg/dL increase |  |  |  |  |
| Model 1 | 0.979 (0.971–0.986) | <0.001 | 0.968 (0.961–0.976) | <0.001 |
| Model 2 | 0.992 (0.983–1.001) | 0.092 | 0.986 (0.977–0.995) | 0.003 |

AIP, atherogenic index of plasma; CACS, coronary artery calcium score; CAD, coronary artery disease; CI, confidence interval; HDL-C, high-density lipoprotein cholesterol; OCP, obstructive coronary plaque; OR, odds ratio

Model 1: unadjusted

Model 2: adjusted for age >60 years, male sex, hypertension, diabetes mellitus, dyslipidaemia, obesity, and proteinuria
